# A Pediatric Covariate Function for CYP3A-Mediated Midazolam Clearance Can Scale Clearance of Selected CYP3A Substrates in Children

**DOI:** 10.1208/s12248-019-0351-9

**Published:** 2019-06-27

**Authors:** Janneke M. Brussee, Elke H. J. Krekels, Elisa A. M. Calvier, Semra Palić, Amin Rostami-Hodjegan, Meindert Danhof, Jeffrey S. Barrett, Saskia N. de Wildt, Catherijne A. J. Knibbe

**Affiliations:** 1 Division of Systems Biomedicine and Pharmacology, Leiden Academic Centre for Drug Research (LACDR), Leiden University, Leiden, The Netherlands; 2 Present Address: Dutch Cancer Institute (NKI), Amsterdam, The Netherlands; 3 Centre for Applied Pharmacokinetic Research, University of Manchester, Manchester, UK; 4 Simcyp Limited (A Certara Company), Sheffield, UK; 5 Bill & Melinda Gates Medical Research Institute, Cambridge, Massachusetts USA; 6 Department of Pediatrics, Division of Clinical Pharmacology & Therapeutics, Children’s Hospital of Philadelphia, Philadelphia, Pennsylvania USA; 7 Department of Pharmacology and Toxicology, Radboud University Medical Centre, Nijmegen, The Netherlands; 8 Intensive Care and Department of Pediatric Surgery, Erasmus MC - Sophia Children’s Hospital, Rotterdam, The Netherlands; 9 Department of Clinical Pharmacy, St. Antonius Hospital, PO Box 2500, 3430 EM Nieuwegein, The Netherlands

**Keywords:** Clearance, CYP3A ontogeny, Scaling function, Pediatrics, Population pharmacokinetics

## Abstract

Recently a framework was presented to assess whether pediatric covariate models for clearance can be extrapolated between drugs sharing elimination pathways, based on extraction ratio, protein binding, and other drug properties. Here we evaluate when a pediatric covariate function for midazolam clearance can be used to scale clearance of other CYP3A substrates. A population PK model including a covariate function for clearance was developed for midazolam in children aged 1–17 years. Commonly used CYP3A substrates were selected and using the framework, it was assessed whether the midazolam covariate function accurately scales their clearance. For eight substrates, reported pediatric clearance values were compared numerically and graphically with clearance values scaled using the midazolam covariate function. For sildenafil, clearance values obtained with population PK modeling based on pediatric concentration-time data were compared with those scaled with the midazolam covariate function. According to the framework, a midazolam covariate function will lead to systemically accurate clearance scaling (absolute prediction error (PE) < 30%) for CYP3A substrates binding to albumin with an extraction ratio between 0.35 and 0.65 when binding < 10% in adults, between 0.05 and 0.55 when binding > 90%, and with an extraction ratio ranging between these values when binding between 10 and 90%. Scaled clearance values for eight commonly used CYP3A substrates were reasonably accurate (PE < 50%). Scaling of sildenafil clearance was accurate (PE < 30%). We defined for which CYP3A substrates a pediatric covariate function for midazolam clearance can accurately scale plasma clearance in children. This scaling approach may be useful for CYP3A substrates with scarce or no available pediatric PK information.

**Electronic supplementary material:**

The online version of this article (10.1208/s12248-019-0351-9) contains supplementary material, which is available to authorized users.

## INTRODUCTION

To define the optimal first-in-child dose during drug development and to develop pediatric dose recommendations for clinical practice, accurate scaling of the plasma clearance of drugs is essential (1–3). This is of particular relevance as performing dedicated pharmacokinetic (PK) studies for all drugs in all pediatric (sub)populations may not be feasible because it would take too many resources. Moreover, this may not even be necessary when other methods are available and could therefore even be considered unethical. One proposed approach shares PK information of drugs eliminated by the same pathway by extrapolating covariate relationships for clearance between drugs (4). This has already been successfully applied for scaling pediatric clearance for drugs glucuronidated by UGT2B7 enzymes and also for drugs eliminated by glomerular filtration (4–6).

Within this context, recently, a framework was presented by Calvier *et al.* for hepatically metabolized drugs identifying the conditions for which between-drug extrapolation is systematically accurate (7). This framework takes into account changes in physiological parameters with age, including changes in (hepatic) blood flow, plasma protein concentrations, hematocrit, liver size, the amount of microsomal protein per gram of liver, and the ontogeny of isoenzyme expression (the microsomal intrinsic clearance) (7). One of the key findings of this framework was that the accuracy of this scaling method depends on the fraction metabolized by the isoenzyme pathway for which plasma clearance is scaled, on the hepatic extraction ratio of both the probe drug and the evaluated drugs in adults, on the type of binding plasma protein, and on the unbound drug fraction (*f*_u_) in adults (7).

As many drugs are eliminated by the cytochrome P450 (CYP) 3A enzyme family (8,9), a pediatric covariate function for CYP3A-mediated clearance may aid in scaling clearance of CYP3A substrates. Midazolam is an established probe drug for CYP3A-mediated clearance (10,11), mainly metabolized by CYP3A4, and to a lesser extent by CYP3A5 (12), and has an intermediate extraction ratio (13). Our aim is to evaluate when a pediatric covariate function for midazolam clearance can be used to scale clearance of other CYP3A substrates in children, taking into account the recent insights of the developed framework (7).

## METHODS

### Overall Approach

A population PK model for midazolam in children was developed based on concentration-time data, to establish a pediatric covariate function for midazolam clearance. Next, we selected a range of drugs that are CYP3A substrates that are commonly prescribed in children, covering compounds prescribed for varying indications in different therapeutic areas, with oral or intravenous administration, and with different drug properties, i.e., alprazolam (14), atorvastatin (15), cisapride (16), domperidone (17), quinidine (18), sildenafil (19), simvastatin (20), solifenacin (21), sufentanil (22), sirolimus (23), tacrolimus (24), tamsulosin (25), and vincristine (26). Based on the drug properties of these CYP3A substrates, we used the framework of Calvier *et al.* (7) to define which age the covariate function for midazolam can be used for accurate scaling of pediatric clearance of the CYP3A substrates from adult clearance values. For eight of the selected CYP3A substrates, pediatric and adult clearance values were available in literature, allowing for the assessment of the accuracy of the scaling function by comparing pediatric clearance values that were scaled from adult clearance values using the covariate function for midazolam to the published literature clearance values in children. Furthermore, for sildenafil, concentration-time data were available from 156 children (27). Using these data, we developed two pediatric population PK models for sildenafil; one using the pediatric covariate function of midazolam clearance directly and one in which the covariate relationship for clearance was optimized using a data-driven analysis, after which, the performance of both models, as well as the estimated and scaled clearance values, was compared.

### Midazolam Population PK Model

Midazolam PK data were available from 31 patients (15 male, 16 female) from the Children’s Hospital of Philadelphia, PA (Table I), with a median age of 8 years of age (range 1–17 years) and a median body weight of 30.2 kg (range 9.5–83.2 kg) (28). Before participation, signed informed consent, by the subject’s parents or guardian, and assent were obtained. Children undergoing surgery were included if they met the criteria I or II of the American Society of Anesthesiologist’s (ASA) classification. A median dose of 12.5 mg (range 3–15 mg) of midazolam was administered as oral suspension (5 mg/mL, Roche Laboratories) to the patients pre-operatively. Blood was densely sampled for midazolam plasma concentrations around 0.25, 0.5, 1, 1.5, 2, 3, 4, 6, 8, 10, and 22 h after dose administration, with a median of 10 samples per patient (range 8–11). Blood was centrifuged and plasma samples stored at < − 20 °C, until midazolam plasma concentrations were determined using LC/MS (28).

**Table I:** Study and Patient Characteristics of the Midazolam and Sildenafil PK Studies

	Midazolam	Sildenafil
Indication	Pre-operatively	Pulmonary arterial hypertension
Number of patients	31	156
Number of samples	327	591
Samples/patient*	10 (8–11)	4 (1–4)
Age (years)*	8 (1–17)	10 (1–17)
Body weight (kg)*	30.2 (9.5–83.2)	28.0 (8.2–106.0)
Male/female, *n* (%)	15/16 (48/52%)	57/99 (37/63%)
Dose (mg)*	12.5 (3–15)	20 (10–80)

*median (range)

A population PK model was developed using non-linear mixed effects modeling (NONMEM version 7.3, ICON, Globomax LLC, Ellicott, MD, USA; Perl-speaks-NONMEM (PsN) version 4.2.0, Uppsala, Sweden; and Pirana 2.9.0, Pirana Software & Consulting BV, Denekamp, the Netherlands) based on first-order conditional estimation with interaction. R (version 3.3.1) and RStudio (version 0.98.1078) were used for data visualization. Several structural models were considered, including 1-, 2-, and 3-compartmental models, and evaluated based on criteria for model stability, goodness-of-fit, and parameter precision, and on comparisons of the objective function values (OFV, − 2 × log-likelihood), using a significance level of *p* < 0.05. The absorption rate could not be estimated and was therefore fixed at 3.5 h^−1^ (29), which results in a *t*_max_ around 0.5 h post-dose, which is in agreement with known values.

Interindividual variability in the estimated parameters for clearance and central volume of distribution was included in the model by the following equation:

1 $$ {P}_i={\theta}_{\mathrm{TV}}\times {e}^{\eta_i} $$

in which *P*_*i*_ is the individual parameter estimate for individual *i*, *θ*_TV_ is the typical value of the parameter in the studied population, and *η*_*i*_ is a random variable for the *i*th individual from a normal distribution with a mean of zero and variance of *ω*^2^, assuming a log-normal distribution for the parameter value in the population.

To describe residual unexplained variability, a proportional error model, an additive error model, and a combination of the proportional and additive error were considered. The *j*th observed concentration of the *i*th individual (*Y*_*ij*_) was modeled according to

2 $$ {Y}_{ij}={C}_{\mathrm{pred}, ij}\times \left(1+\varepsilon {1}_{ij}\right)+\varepsilon {2}_{ij} $$

where *C*_pred*,ij*_ is the *j*th predicted midazolam concentration of the *i*th individual, and *ε*_*ij*_ is a random variable from a normal distribution with a mean of zero and variance of *σ*^2^, with *ε*1 the proportional error and *ε*2 the additive error.

A systematic covariate analysis was performed for the estimated model parameters in which age, body weight, and sex were tested for statistical significance. For sex, the typical value (*θ*_TV_) for girls was estimated relative to the value for boys. The remaining continuous covariates body weight and age were tested using a power (Eq. 3) function:

3 $$ {P}_i={\theta}_{\mathrm{TV}}\times {\left(\frac{{\mathrm{COV}}_i}{\mathrm{COV}\mathrm{med}}\right)}^{\theta_{\mathrm{COV}}}\times {e}^{\eta_i} $$

where *P*_*i*_ is the individual parameter estimate for individual *i* with a covariate value of COV_*i*,_ *θ*_TV_ is the parameter value for a typical individual with a median covariate value (COV_med_), *θ*_COV_ is the estimated exponent, and *η*_*i*_ is a random variable as described above (Eq. 1). For the forward inclusion of a covariate, a drop in OFV by at least 6.64 points (*p* < 0.01) was considered statistically significant, while for the backward deletion a more stringent *p* value (*p* < 0.005, *Δ*OFV > 7.88) was used. In addition, the interindividual variability in the PK parameter or the residual variability should decrease for a covariate to be retained in the model.

To evaluate whether the model described the observed concentrations well, goodness-of-fit plots were assessed. These diagnostic plots include observed versus population- and individual-predicted concentrations and conditional weighted residuals (CWRES) versus population-predicted concentrations and versus time. To evaluate model stability and parameter precision, a bootstrap analysis (*n* = 250) was performed. Finally, a normalized prediction distribution error (NPDE) analysis was performed using the NPDE package in R (30), with *n* = 1000 simulations to evaluate whether the model can accurately predict the concentration and capture the observed variability.

### Between-Drug Extrapolation Potential of Midazolam Clearance to Other CYP3A Substrates

The previously published framework on between-drug extrapolation of covariate functions (7) was used to assess, based on the drug properties of CYP3A substrates, whether between-drug extrapolation of the covariate relationship for midazolam would lead to accurate scaling of the pediatric clearance of the selected CYP3A substrates. For this, the relevant drug properties, i.e., the extraction ratio, the plasma protein to which the drug is binding, and the *f*_u_ for midazolam and the selected drugs were obtained from literature. In this analysis, the selected drugs were assumed to exclusively bind to either human serum albumin (HSA) or α1-acid glycoprotein (AAG), while midazolam was assumed to bind to either HSA (for comparison with HSA-binding drugs) or to AAG (for comparison with AAG-binding drugs).

Using the extraction ratio and the *f*_u_ of the selected CYP3A substrates that were considered within the results from the framework (7), it was assessed to what age clearance scaling with the covariate function of midazolam would certainly be accurate for the selected drugs. Drugs were selected of which it has been reported that CYP3A is the “major” pathway for elimination, and we assumed CYP3A metabolism to be responsible for ≥ 75% of the total metabolism for both midazolam and all selected substrates. Based on the extraction ratio and *f*_u_ from midazolam, we also derived general criteria for systematically accurate clearance scaling for CYP3A substrates using the covariate function for midazolam clearance according to the framework.

### Comparison of Scaled Versus Reported Pediatric Clearance Values

For the selected CYP3A substrates for which both pediatric and adult clearance values were reported in literature, we applied the pediatric covariate function for midazolam clearance to the reported adult clearance values to scale for pediatric clearance values. For this we assumed that typical adults have a body weight of 70 kg. We graphically compared the scaled typical clearance values with the reported pediatric clearance values. Moreover, we calculated the prediction error (PE) for three typical subjects (an infant of 10 kg, a child of 20 kg, and an adolescent of 50 kg) based on literature values for pediatric clearance using Eq. 4:

4 $$ \mathrm{PE}=\frac{{\mathrm{CL}}_{\mathrm{scaled}}-{\mathrm{CL}}_{\mathrm{ref}}}{{\mathrm{CL}}_{\mathrm{ref}}}\times 100\% $$

with CL_scaled_ the scaled clearance value and CL_ref_ the reported pediatric clearance. An absolute PE of < 30% was considered accurate, an absolute PE of 30–50% reasonably accurate, and an absolute PE of ≥ 50% inaccurate.

### Sildenafil Population PK Models

Sildenafil PK data from a previously published study (27) were made available by Pfizer Inc. In this study, sildenafil PK data were collected from 156 (57 male, 99 female) patients in a randomized, double-blind, placebo-controlled, dose ranging, parallel group study of oral sildenafil for the treatment of children with pulmonary arterial hypertension (27,31). Subjects included children ranging in age from 1 to 17 years (median 10 years), with a median body weight of 28.0 kg (range 8.2–106 kg) (Table I). A median of four samples per patient (range 1–4) was available, with a total of 591 measurements available for analysis. Samples were taken at steady-state at trough and around three, six, and eight hours post-dose. Patients were randomly assigned to a low-, medium-, or high-dose group (*n* = 39, *n* = 48, and *n* = 69, respectively), and the dosages were weight-stratified, with a medium dose of 10, 20, and 40 mg and a high dose of 20, 40, and 80 mg for patients of 8–20 kg, 20–45 kg or > 45 kg, respectively. The low dose was 10 mg for all patients > 20 kg, and patients with a body weight ≤ 20 kg received either a medium or high dose, as no drug effect was expected with a lower dose than 10 mg (27,31). In the population PK analysis, the samples without recorded sampling times were excluded.

Based on these data, a “reference model” was developed in the same manner as described for midazolam. The absorption rate constant could not be estimated and was therefore fixed at 1 h^−1^, leading to a maximum concentration around two hours post-dose, which was before the first sample was taken.

The extrapolation potential of the covariate function for midazolam clearance was evaluated in a second population PK model referred to as the “extrapolation model.” This model was kept the same as the reference model, except the clearance was not estimated, but scaled from an apparent CL/F value of 100 L/h for adults, which was derived from reported systemic clearance and oral bioavailability values of 41 L/h and 0.41 L/h, respectively (32), using the covariate function for midazolam clearance. We assumed the same bioavailability in adults and pediatric patients.

The reference and extrapolation models were evaluated in the same manner as the midazolam PK model (see under “Midazolam Population PK Model”).

Sildenafil clearance values from the sildenafil the “reference model” (CL_ref_) and the sildenafil “extrapolation model” (CL_scaled_) were compared graphically. For a numerical comparison of both sildenafil models, typical clearance values for three typical subjects (an infant of 10 kg, a child of 20 kg, and an adolescent of 50 kg) were calculated, and a PE for clearance was calculated using Eq. 4.

## RESULTS

### Midazolam Population PK Model

For midazolam, a two-compartmental model with body weight, included in an exponential covariate relationship on clearance, volumes of distribution, and intercompartmental clearance, best described the data (Table II, fig. S2, S3). As midazolam was administered orally, apparent parameters for clearance and volume of distribution were obtained. For a typical individual of 30.2 kg, apparent clearance was 102.6 L/h, and the exponent, in the exponential equation relating body weight and clearance, was found to be 0.874 (Table II). As a result, this pediatric covariate function was used to scale CYP3A-mediated clearance in the between-drug extrapolation:

5 $$ {\mathrm{CL}}_{\mathrm{pediatric}}={\mathrm{CL}}_{\mathrm{adult}}\times {\left(\frac{\mathrm{WT}}{70}\right)}^{0.874} $$

**Table II:** Model Parameter Estimates for the Midazolam PK Model and the Bootstrap Results Based on *n* = 250 Resampling

Parameter		Model estimate (RSE)	Bootstrap median (90 CI)
Midazolam clearance^**†**^ CL_*i*_ = CL_30.2 kg_ × (WT_*i*_/30.2)^k1^	(CL/F)_30.2 kg_ (L/h)	102.6 (9%)	101.4 (89.1–118.2)
	*k*1	0.874 (13%)	0.901 (0.698–1.11)
Volume of distribution^**†**^ *V*_c,*i*_ = *V*_c,30.2 kg_ × (WT_*i*_/30.2)^k2^	(V_c_/F)_30.2 kg_ (L)	156 (25%)	141 (76.5–210)
	*k*2	1.88 (17%)	2.15 (1.43–3.30)
Peripheral volume^**†**^ *V*_p,*i*_ = *V*_p,30.2 kg_ × (WT_*i*_/30.2)^k3^	(V_p_/F)_30.2 kg_ (L)	255 (14%)	252 (197–338)
	*k*3	0.91 (23%)	0.88 (0.60–1.21)
Intercompartmental clearance^**†**^ *Q*_*i*_ = *Q*_30.2 kg_ × (WT_*i*_/30.2)^k4^	(Q/F)_30.2 kg_ (L/h)	121.8 (21%)	115.6 (73.7–163)
	*k*4	0.75 fix	0.75 fix
Absorption rate constant	*k*_a_ (h^−1^)	3.5 fix	3.5 fix
IIV clearance	*ω*^2^ CL/F	0.158 (41%)	0.145 (0.063–0.259)
IIV volume of distribution	*ω*^2^ *V*_c_/F	1.19 (27%)	1.06 (0.64–1.71)
Proportional error	*σ* ^2^	0.283 (13%)	0.272 (0.222–0.346)

RSE is the relative standard error, and 90 CI is the 90% confidence interval representing the 5th and 95th percentiles. Interindividual and residual variability values are shown as variance estimates

^†^Parameters are apparent parameters, as only oral data was included

### Between-Drug Extrapolation Potential of Midazolam Clearance to Other CYP3A Substrates

**Fig. 1:**
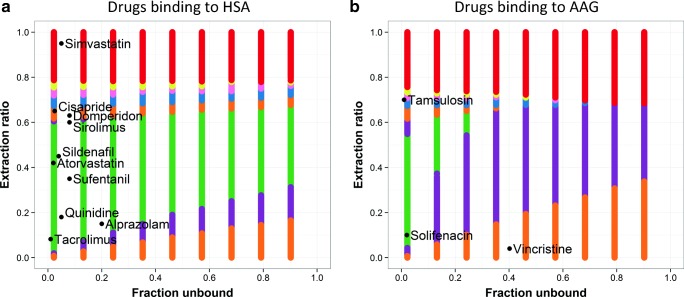
Prediction of the age down to which the pediatric covariate function for CYP3A-mediated midazolam clearance can be used to accurately scale clearance of CYP3A substrates with specific drug properties. Extraction ratio values of the tested CYP3A substrates are plotted versus the fraction unbound (*f*_u_) of the test drugs in adults. The color scheme was obtained from the published framework (7) and represents hypothetical model-test drug combinations that lead to systematically accurate scaling of clearance in children down to 1 day (green), 1 month (purple), 6 months (orange), 1 year (blue), 2 years (pink), and 5 years of age (yellow). Red indicates that scaling is not systematically accurate in children of 5 years and younger. The black data points represent the included test drugs and their extraction ratio and *f*_u_ values. Panel a shows drugs binding to albumin (HSA), while panel b shows drugs binding to α1-acid glycoprotein (AAG). Modified from Calvier *et al.* (7) (with permission)

The obtained drug properties of midazolam and the selected CYP3A substrates required for between-drug extrapolation of clearance are listed in Table SI (33–54). Figure S1 shows down to what age the clearance of the selected substrates can at least be extrapolated from adult values with the covariate relationships for midazolam clearance, based on the differences in extraction ratio and *f*_u_ according to the framework that was previously reported (7). Based on this information, Fig. 1 was derived showing when scaling of pediatric clearance of a CYP3A substrate will be accurate depending on its extraction ratio and *f*_u_ values in adults. This figure shows that this method will accurately scale pediatric clearance values down to neonates of 1 day of age for alprazolam, atorvastatin, quinidine, sildenafil, solifenacin, sufentanil, and tacrolimus, while for the other drugs clearance will be at least accurately scaled down to infants of 1 month (sirolimus) and 6 months of age (cisapride, domperidone, and vincristine). Tamsulosin clearance scaling will be accurate down to at least 2 years of age, while for simvastatin accurate scaling down to 5 years of age may not even be possible (Fig. 1).

From Fig. 1 it can also be derived that a pediatric covariate function for midazolam can be used to scale CYP3A-mediated clearance across all ages including neonates (i.e., green bars) of HSA-bound substrates which are highly protein bound (> 90%, *f*_u_ ≤ 0.1), provided the extraction ratio in adults ranges between 0.05 and 0.55. Similarly, for HSA-bound substrates with low protein binding (< 10%, *f*_u_ ≥ 0.9), the drug to which the covariate function can be extrapolated should have an extraction ratio between 0.35 and 0.65. In between these extreme percentages of binding to HSA, the required extraction ratio gradually changes between these values (green bars, Fig. 1). For AAG-bound drugs, fewer combinations of drug properties lead to accurate scaling based on a midazolam pediatric covariate function, with no scenarios for drugs with low or intermediate protein binding (< 60%, *f*_u_ ≥ 0.4), while an extraction ratio of 0.4–0.6 or 0.1–0.5 in adults leads to accurate scaling for drugs that are around 90% or ≥ 97.5% bound, respectively (Fig. 1).

### Comparison of Scaled Versus Reported Pediatric Clearance Values

**Fig. 2:**
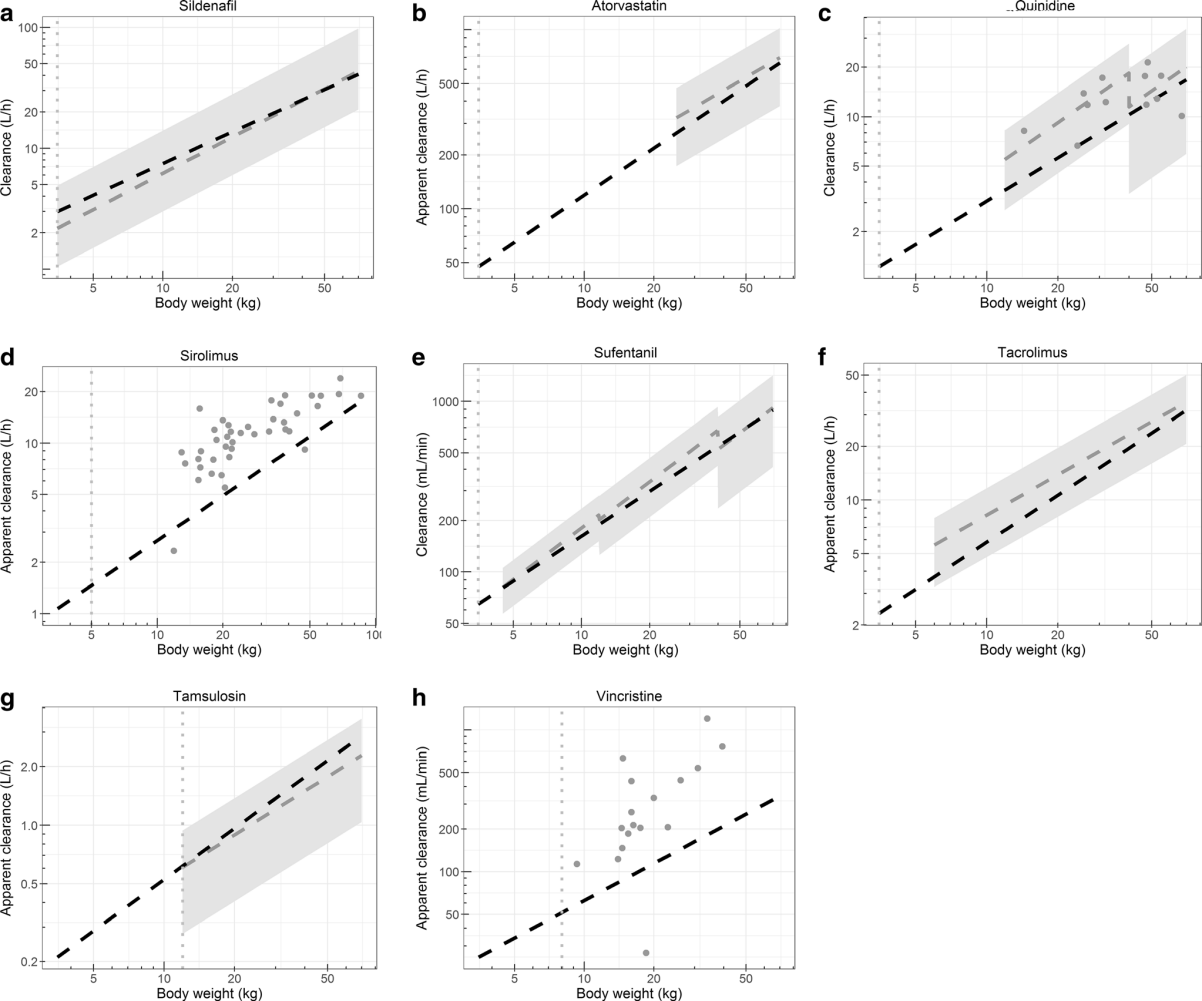
Scaled and reported clearance values versus body weight for various CYP3A substrates. Clearance (or apparent clearance) values are based on the between-drug extrapolation of the pediatric covariate function for CYP3A-mediated midazolam clearance and reported adult clearance values (black) and based on reported pediatric clearance values in literature (gray), for sildenafil (**a**), atorvastatin (**b**), quinidine (**c**), sirolimus (**d**), sufentanil (**e**), tacrolimus (**f**), tamsulosin (**g**), and vincristine (**h**). The vertical dotted line (gray) indicates the body weight down to which systemically accurate clearance scaling is predicted to be possible according to the framework (7). For the reported clearance values the following is depicted: **a** Mean sildenafil clearance (line) with minimal and maximal reported values (gray area). **b** Typical atorvastatin clearance (line) ± 46.3% (%CV, gray area). **c** Mean quinidine clearance (line) ± 2 SD (gray area) and individual-reported clearances (closed circles). **d** Individual-reported sirolimus clearances (closed circles). **e** Mean sufentanil clearance (line) ± 2 SD (gray area). **f** Typical tacrolimus clearance (line) ± 41.6% (%CV, with $\mathrm{CV}=\sqrt{e^{\sigma^2}-1}$, and *σ*^2^ = 0.16, gray area). **g** Typical tamsulosin clearance (line) ± 54.4% (%CV, gray area). **h** Individual-reported vincristine clearances, corrected for body surface area (closed circles)

Obtained pediatric and adult clearance values of CYP3A substrates are summarized in Table SI (32,35,55–65). In Fig. 2, the scaled clearance values are shown together with the reported pediatric clearance values for the various substrates versus body weight. Table IV lists the calculated prediction errors for the three typical pediatric individuals. For most drugs, the scaled covariate relationships fall within the range of observed values, except for vincristine and sirolimus. The calculated PE values also show that scaled vincristine and sirolimus clearance values are inaccurate; although with a PE value of 64.3% and 58.3%, respectively, this inaccuracy is not extreme. The PE values for all other drugs are < 50%, indicating accurate or reasonably accurate scaling of clearance in infants, children, and adolescents.

### Sildenafil Population PK Models

The reference model and extrapolation model for sildenafil described the sildenafil concentrations with a one-compartmental model. Table III presents model parameters and bootstrap values for both models and the goodness-of-fit plots and results from the NPDE analyses are presented in fig. S4 and fig. S5, respectively. These results show that descriptive and predictive properties of both models are similar.

**Table III:** Model Parameter Estimates for the Sildenafil “Reference Model” Versus the Sildenafil “Extrapolation Model” and the Bootstrap Results for both Models Based on *n* = 250 Resampling

Parameter		Reference model		Extrapolation model	
		Model estimate (RSE)	Bootstrap (90 CI)	Model estimate (RSE)	Bootstrap (90 CI)
Sildenafil clearance^**†**^ CL_*i*_ = CL_70 kg_ × (WT_*i*_/70)^k1^	(CL/F)_70 kg_ (L/h)	113 (13%)	112 (84.6–149)	100 fix	100 fix
	*k*1	1.08 (11%)	1.05 (0.82–1.30)	0.874 fix	0.874 fix
Volume of distribution^**†**^ *V*_*i*_ = *V*_28 kg_ × (WT_*i*_/28)^k2^	(V/F)_28 kg_ (L)	540 (33%)	561 (311–1424)	590 (29%)	574 (389–1134)
	*k*2	3.18 (10%)	3.17 (2.41–4.27)	3.18 (10%)	3.16 (2.49–4.01)
Absorption rate constant	*k*_a_ (h^−1^)	1 fix	1 fix	1 fix	1 fix
IIV clearance	*ω*^2^ CL/F	0.493 (14%)	0.487 (0.363–0.631)	0.510 (13%)	0.512 (0.397–0.650)
Proportional error	*σ* ^2^	0.627 (7%)	0.616 (0.538–0.703)	0.651 (8%)	0.646 (0.564–0.738)

RSE is the relative standard error, and 90 CI is the 90% confidence interval representing the 5th and 95th percentiles. Interindividual and residual variability values are shown as variance estimates

^†^Parameters are apparent parameters, as only oral data was included

**Fig. 3:**
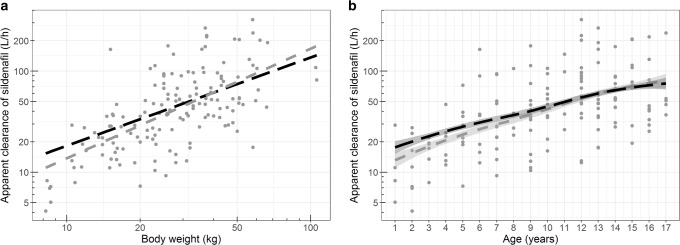
Sildenafil apparent clearance versus body weight (**a**) and versus age (**b**) for the sildenafil reference model (gray) and based on between-drug extrapolation of clearance (black) with points representing the individual-predicted clearance by the reference model. In panel a, the lines represent population-predicted clearance values directly derived from the body weight–based covariate relationship, while in panel b, the lines represent the loess function summarizing the population-predicted clearance values with a 95% confidence interval (shaded area)

In the reference model, apparent sildenafil clearance for a typical individual of 28 kg was found to be 41.9 L/h, and clearance increased exponentially with increasing body weight (exponent of 1.08 [RSE 11%]), leading to an apparent clearance of 113 L/h for a 70-kg individual. In the extrapolation model, apparent clearance was scaled using Eq. 5, with a CL_adult_ of 100 L/h for a 70-kg individual, leading to a scaled apparent clearance of 44.9 L/h for a 28-kg individual. As shown in a graphical comparison in Fig. 3a, both covariate relationships are very similar, with only a small difference in clearance values between the two models for children with the lowest body weight. Figure 3 b shows that when individual clearance predictions by both models are plotted versus age, the loess function for these relationships is also similar with the small difference in the youngest age range. These small discrepancies may be due to the small number of individuals in the youngest age group (1–2 years of age) in the population receiving midazolam used for establishing the pediatric covariate function.

The PE for clearance increases with a decreasing age, with a PE of − 5.2%, 14.6%, and 32.1%, in an adolescent, child, and infant, respectively, indicating that with a decreasing age and body weight, the extrapolation model yields a larger overprediction of clearance. However, the scaled clearance values are within the range of observed clearance values, which show a high variability throughout the pediatric age range (Fig. 3b).

## DISCUSSION

Accurate scaling of plasma clearance is essential to establish optimal first-in-child doses during drug development and for the development of pediatric dose recommendations. As many drugs are metabolized by CYP3A enzymes and midazolam is a commonly accepted probe drug for CYP3A, we aimed to evaluate when a pediatric covariate function for CYP3A-mediated midazolam clearance can be used to scale pediatric clearance of CYP3A substrates given the recently reported guidance on between-drug extrapolation of covariate models.

Whether in this case, scaling of pediatric clearance of CYP3A substrates based on a covariate function from a pediatric population PK model for midazolam is accurate may depend on the drug properties as was reported by Calvier *et al.* before (7). We used this previously developed framework (7) to assess for which of the selected CYP3A substrates scaling with the pediatric covariate function from midazolam will lead to accurate clearance values (Fig. 1). The color code in Fig. 1 indicates down to which age scaling of clearance is expected to be systemically accurate based on the extraction ratio and *f*_u_ in adults. Each colored dot in this graph represents multiple drugs with differences in the remaining drug properties (i.e., blood-to-plasma partitioning and affinity to isoenzymes) and it should be noted that when the framework predicts that scaling of clearance is not systemically accurate for all drugs with the indicated combination of drug properties, there may still be drugs within the set of drugs represented by a data point for which this scaling is accurate. In those cases, it can however not be predicted a priori whether this will be the case for each of the individual drugs (7).

For the selected CYP3A substrates alprazolam, atorvastatin, cisapride, domperidone, quinidine, sildenafil, solifenacin, sufentanil, sirolimus, tacrolimus, and vincristine, based on differences in *f*_u_ and extraction ratio in comparison with midazolam, scaling of clearance with the covariate function of midazolam is expected to be accurate down to children of at least 1 year of age and for some drugs even to neonates and infants (Fig. 1).

Several approaches and methods for scaling of clearance in children have been described in literature, including scaling of clearance using a body weight–based exponential function with exponents of, e.g., 0.67, 0.75, or 1. While some studies showed that allometric scaling may be a reasonable approach (66,67) and other studies disagreed (68,69), in a systematic assessment of the applicability of body weight–based scaling with a fixed exponent of 0.75, it was found that this approach leads to increasingly inaccurate scaled values with decreasing age, reaching prediction errors of up to 278% in neonates (70). Also, other phenotyping studies have used probe drugs to predict clearance of a drug sharing its elimination pathway (71–74). These phenotyping studies include studies applying the “cocktail” approach with five drugs reflecting clearance by five CYP enzymes including CYP3A, which has been considered to be predictive of drug-drug interactions with regard to these enzymes (75), but there is no data on how this approach may predict pediatric clearance. The concept of using probe drugs for drug clearance in the pediatric population by extrapolating pediatric covariate functions for clearance for drugs sharing elimination pathways was therefore developed, and this method had already been successful in scaling pediatric clearance for UGT2B7 substrates and for drugs eliminated through glomerular filtration (4–6). Later, a systematic assessment of this method defined the prerequisites for systematically accurate scaling with this technique (7). In the current work, we illustrate how the knowledge obtained in that analysis can be applied. Moreover, we add CYP3A metabolism to the list of elimination pathways for which between-drug extrapolation of pediatric covariate relationships for clearance has been successfully applied.

Between-drug extrapolation of clearance on the basis of a pediatric covariate function for CYP3A-mediated midazolam clearance, indeed, led to accurate or reasonably accurate scaling of pediatric clearance of most of the selected CYP3A substrates in children (Table IV, Fig. 2). The pediatric covariate function for midazolam clearance can accurately scale pediatric clearance of CYP3A substrates down to at least 1 year of age for a large number of relevant substrates including sildenafil, atorvastatin, quinidine, sufentanil, tacrolimus, and tamsulosin. This indicates accurate predictions for 75% (6 out of 8) of the evaluated compounds. In addition to reported clearance values, for sildenafil, concentration-time data were available as well. With these data, it was further confirmed that the between-drug extrapolation of the covariate relationship of midazolam clearance yields accurate clearance predictions.

**Table IV:** Prediction error (PE) of scaled clearance values using the pediatric covariate function for CYP3A-mediated midazolam clearance versus reported pediatric clearance values for three representative pediatric subjects of 10, 20, and 50 kg (Eq. 4), with negative and positive values for under- and overpredicted clearance values, respectively

	Infant (10 kg)	Child (20 kg)	Adolescent (50 kg)
Atorvastatin	− **26.7%**	− **20.1%**	− **10.5%**
Quinidine	− ***33.5%***	− ***39.0%***	− **12.8%**
Sildenafil	**20.7%**	**10.6%**	− **1.4%**
Sirolimus	NA	− *58.3%*	− **31.5%**
Sufentanil	− **10.3%**	− **12.0%**	**1.1%**
Tacrolimus	− ***44.6%***	− ***39.6%***	− ***32.3%***
Tamsulosin	NA	**8.1%**	**21.2%**
Vincristine	− *64.3%*	NA	NA

NA denotes no pediatric or adult clearance values reported in literature

Colors indicate an accurate prediction (absolute PE < 30%, bold), a reasonably accurate prediction (absolute PE 30–50%, bold italics), and an inaccurate prediction (absolute PE ≥ 50%, italics)

Contrary to what was expected based on the theoretical framework, scaled clearance values of sirolimus and vincristine (2 out of 8 evaluated compounds) were inaccurate compared with reported literature values (PE > 50%, Table IV). For sirolimus, this may be due to the known induction of hepatic CYP3A activity and possibly altered hepatic P-glycoprotein expression (76). The impact of hepatic transporters was not taken into consideration in the framework by Calvier *et al.*, because the impact of these transporters on clearance and their maturation patterns in children remains largely unknown. The scaling of vincristine may be inaccurate, because it is predominantly metabolized by CYP3A5 (77), with a relative smaller contribution of CYP3A4-mediated metabolism compared with midazolam, while midazolam is mainly metabolized by CYP3A4 (12). No pharmacogenomics data on CYP3A polymorphisms were collected, which could have explained some of the observed interindividual variability in clearance. Other factors that may affect the accuracy of our pathway-specific scaling approach, apart from the hepatic extraction ratio and *f*_u_ in adults, include that the fraction eliminated by a certain pathway may be different from the ≥ 75% assumed here. It has for instance been shown that the age down to which clearance can accurately be scaled increases when the contribution of CYP3A metabolism to the overall hepatic metabolism is decreasing (7). Additionally, the contribution of minor elimination pathways to overall drug clearance has been ignored in the current analysis. Moreover, scenarios for the between-drug extrapolation of pediatric covariate functions for clearance of HSA-bound drugs to AAG-bound drugs have not been investigated; therefore, we assumed midazolam to be AAG-bound when using its covariate function to scale the clearance of AAG-bound CYP3A substrates. The impact of this assumption would be largest in neonates and in the youngest children < 1 year of age; as in these age groups, the concentration of AAG is known to vary more with age than the concentration of HSA, due to the fact that AAG concentrations take longer time to mature and reach adult levels (78). It should also be taken into account that stress and disease state may impact protein binding and thereby alter the unbound fraction within an individual over time (79,80). Lastly, as in the sildenafil PK study, not many samples were taken shortly after administration; absorption rate constants for sildenafil could not be estimated and were therefore fixed at 1 h^−1^, which is between the reported values for *k*_a_ of 0.34 h^−1^ (81) and 4.51 h^−1^ (82). A sensitivity analysis showed that fixing it at different values had no impact on the scaled clearance values.

In this analysis, we only included midazolam PK data from children > 1 year of age, and therefore, the pediatric covariate function for midazolam clearance we developed in this analysis cannot be used to scale clearance values of CYP3A substrates in neonates and infants < 1 year of age. Extrapolation of the covariate relationship to (preterm) neonates and infants is anticipated to yield overprediction of clearance, as CYP3A-mediated metabolism in this young age group is lower due to the large impact of maturation in the first weeks and months of life (83). To be able to apply this covariate function to scale CYP3A-mediated clearance in neonates and young infants up to 1 year of age, the model should be extended with a covariate relationship for clearance based on data from children < 1 year of age.

## CONCLUSION

This analysis showed that a pediatric covariate relationship describing how midazolam clearance changes throughout the pediatric age range can be used to scale adult clearance values for many other CYP3A substrates to pediatric clearance values. Specifically, it was found that this approach is applicable to accurately scale clearances of drugs that are mainly eliminated by CYP3A-mediated metabolism with for example high protein binding to HSA (> 90%) and a low to intermediate extraction ratio of < 0.55 in adults. The possibility to scale clearance of CYP3A substrates with the appropriate properties in children from adult clearance using a pediatric covariate function for CYP3A-mediated midazolam clearance may improve pediatric dosing guidelines of CYP3A substrates for clinical practice and may aid in determining the dose in first-in-child studies involving new CYP3A substrates depending on its drug properties. This may be especially useful for CYP3A substrates in which scarce or no pediatric PK information is available, for example for alprazolam, domperidone, and solifenacin.

## Electronic supplementary material

ESM 1 (DOCX 1.69 mb)
