# Supplementary material for: A Pediatric Covariate Function for CYP3A-Mediated Midazolam Clearance Can Scale Clearance of Selected CYP3A Substrates in Children
Source: AAPS J. 2019 Jun 27;21(5):81. doi: 10.1208/s12248-019-0351-9 (PMC6597607; doi:10.1208/s12248-019-0351-9)
Supplement: Supplementary file 1 — (DOCX 1.69 mb) [file 12248_2019_351_MOESM1_ESM.docx]

**Electronic Supplementary Material**

**A Pediatric Covariate Function for CYP3A-Mediated Midazolam Clearance Can Scale Clearance of Selected CYP3A Substrates in Children**

Brussee JM, Krekels EHJ, Calvier EAM, Palić S, Rostami-Hodjegan A, Danhof M, Barrett JS,
De Wildt SN, Knibbe CAJ

Contact: [c.knibbe@antoniusziekenhuis.nl](mailto:c.knibbe@antoniusziekenhuis.nl)

**Table SI.** Drug properties and reported clearance values of selected CYP3A substrates

| Drug | ER_adult_ | Ref | Protein binding | Ref | F_u,adult_ | Ref | Reported CL_adult,70kg_ | Ref | Reported CL_child_ | Ref | Age range” |
| --- | --- | --- | --- | --- | --- | --- | --- | --- | --- | --- | --- |
| Midazolam | 0.354 | (33) | HSA | (35) | 0.022 | (34) | - |  | - |  | - |
| Alprazolam | 0.15 | (35) | HSA | (35) | 0.20 | (35) | - |  | - |  | - |
| Atorvastatin | 0.42 | (36) | HSA | (36) | 0.02 | (35, 37) | 652 L/h^#^ | (55) | $699\cdot\left( \frac{WT}{70} \right)^{0.75}$L/h^#^ | (55) | 6-17 y |
| Cisapride | 0.65 | (35) | HSA | (38) | 0.025 | (38) | - |  | - |  | - |
| Domperidone | 0.63 | (39) | HSA | (39) | 0.08 | (39) | - |  | - |  | - |
| Quinidine | 0.18 | (40) | HSA | (41) | 0.05* | (37) | 4 mL/min/kg | (35) | Individual values,  0.461 L/h/kg  0.287 L/h/kg^+^ | (62) | 3.7-12 y  12-22 y |
| Sildenafil | 0.45 | (42) | HSA | (43) | 0.04 | (43) | 41 L/h | (32) | 0.62 L/h/kg | (61) | 0.5-10 y |
| Simvastatin | 0.95 | (44) | HSA | (36) | 0.05 | (35, 37) | - |  | - |  | - |
| Sirolimus | 0.60 | (45) | HSA | (46) | 0.08 | (37) | 0.210 L/h/kg^#^ | (56) | Individual values | (63) | 3-18 y |
| Solifenacin | 0.10 | (47) | AAG | (47) | 0.02 | (37, 47) | - |  | - |  | - |
| Sufentanil | 0.35 | (48) | HSA | (49) | 0.08 | (37) | 0.762 L/h/kg | (57) | 18.1 mL/min/kg 16.9 mL/min/kg 13.1 mL/min/kg**^†^** | (64) | 1 m - 2 y 2-12 y 12-18 y |
| Tacrolimus | 0.082 | (50) | HSA | (51) | 0.01 | (35, 37) | 31.8 L/h^ǂ#^ | (58) | $12.9\cdot\left( \frac{WT}{13.2} \right)^{0.75}$L/h^#^ | (65) | 0.65-17 y |
| Tamsulosin | 0.70^^^ | (52) | AAG | (52) | 0.01 | (37, 52) | 2.88 L/h | (59) | $2.28\cdot\left( \frac{WT}{70} \right)^{0.75}$L/h^#^ | (59) | 2-16 y |
| Vincristine | 0.04 | (53) | AAG | (54) | 0.402 | (54) | 0.293 L/h/kg | (60) | Individual values, 1.049 L/h/kg | (60) | 1.3-12.4 y |

ER: extraction ratio. F_u_: fraction unbound. Ref: reference.

HSA: human serum albumin. AAG: α1-acid glycoprotein

”Age range of pediatric subjects in the study where pediatric clearance is reported, with age in years (y) or months (m).

^#^Reported clearances are apparent clearances (CL/F).

*Brocks *et al.* describe a different value of f_u_ of 0.23 (86), but both f_u_ values (0.23 or 0.05) lead to the same conclusion down to which age accurate scaling is possible (down to ≤ 1 day of age).

^+^Quinidine clearance in children was reported for children 3.7-12 years and ≥12 years of age.

**^†^**Sufentanil clearance in children was reported for 3 different age groups of 1-24 months of age, 2-12 years of age, and 12-18 years of age, with assumed weight ranges (based on WHO guidelines) of 4.5-12, 12-40 and 40-70 kg respectively.

^ǂ^Tacrolimus clearance was derived from dose and exposure.

^^^Vincristine is reported to be a high extraction ratio drug. Therefore, the extraction ratio was assumed 0.7, but this may be higher.

A. Drugs binding to HSA B. Drugs binding to AAG


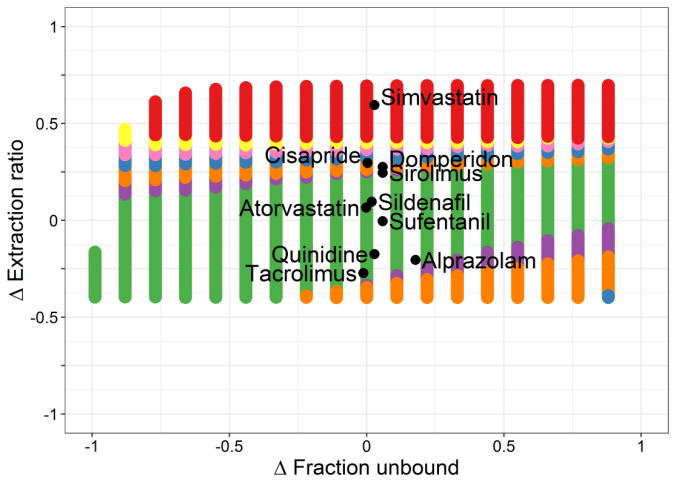

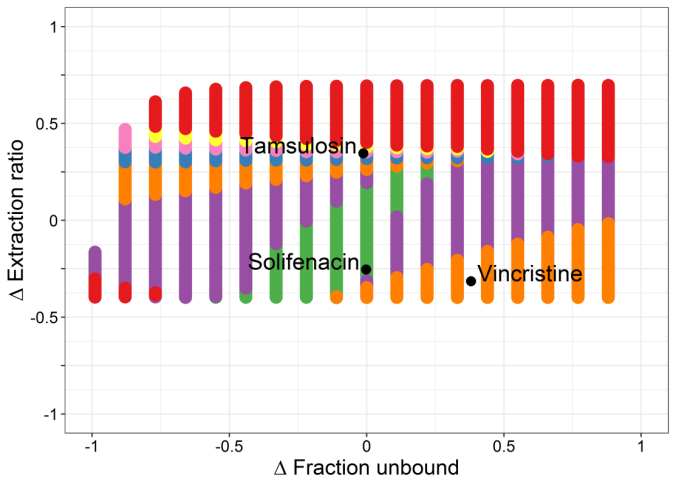


**Figure S1**. Prediction of the age down to which the pediatric covariate function for CYP3A-mediated midazolam clearance can be used to accurately scale clearance of CYP3A substrates with specific drug properties. Difference in extraction ratio between CYP3A substrates (test drugs) and midazolam (model drug) is plotted versus difference in fraction unbound (f_u_) between these drugs. The color scheme was obtained from the published framework (7) and represent hypothetical model-test drug combinations that lead to systematically accurate scaling of clearance in children down to 1 day (green), 1 month (purple), 6 months (orange), 1 year (blue), 2 years (pink), and 5 years of age (yellow). Red indicates that scaling is not systematically accurate in children of 5 years and younger. The black data points represent the included test drugs and their parameter values relative to midazolam. Panel A shows drugs binding to albumin (HSA), while panel B shows drugs binding to α1-acid glycoprotein (AAG). Modified from Calvier *et al*. (7) (with permission).

A B


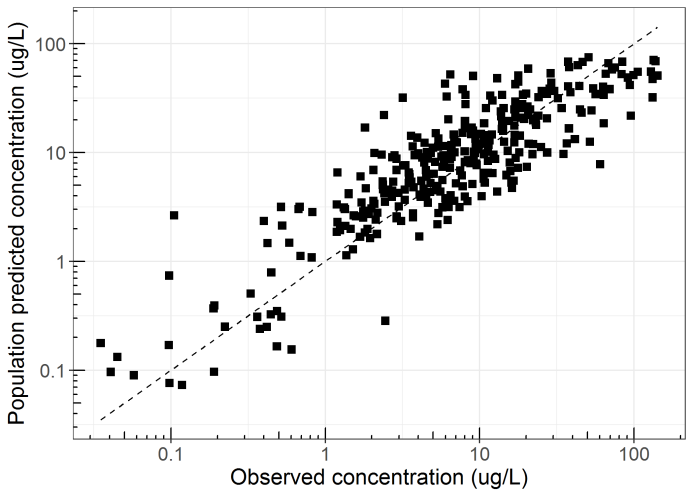

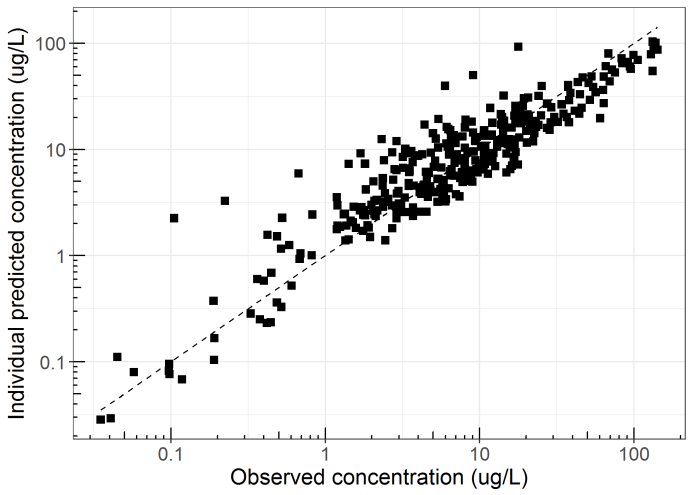


C D

**
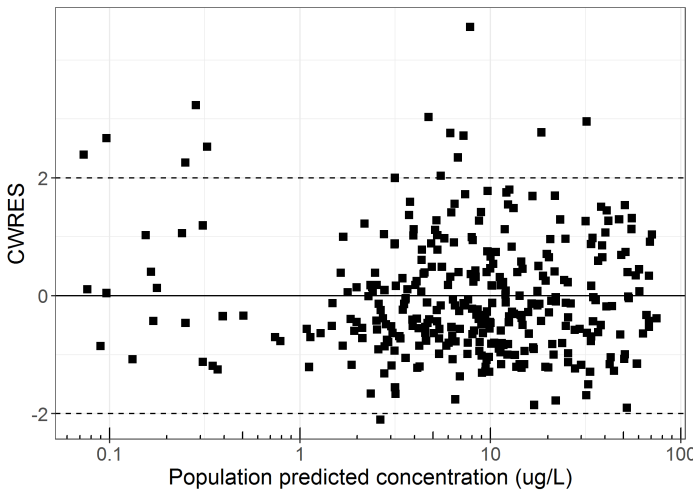

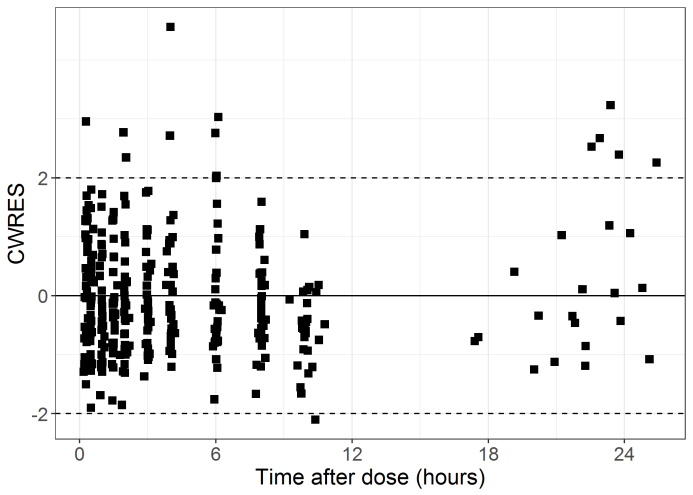
**

**Figure S2.** Goodness-of-fit plots for the pediatric population PK model of midazolam (model drug). Plots include individual and population predicted concentration versus observed concentration (A,B) and conditionally weighted residuals (CWRES) versus predicted concentration (C) and versus time after dose (D).

A


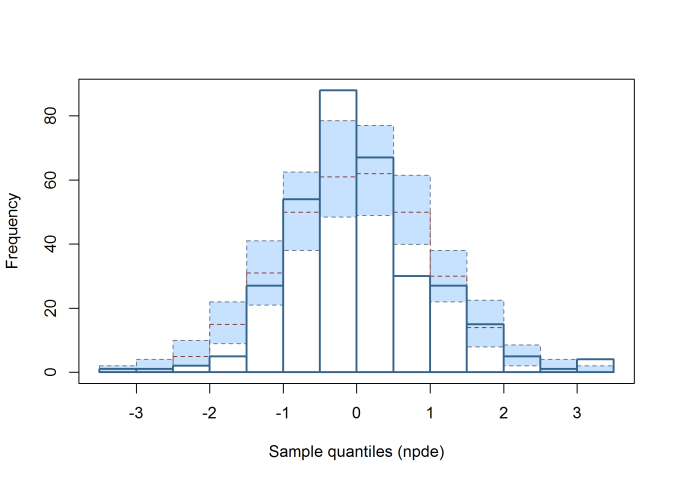


B C

**
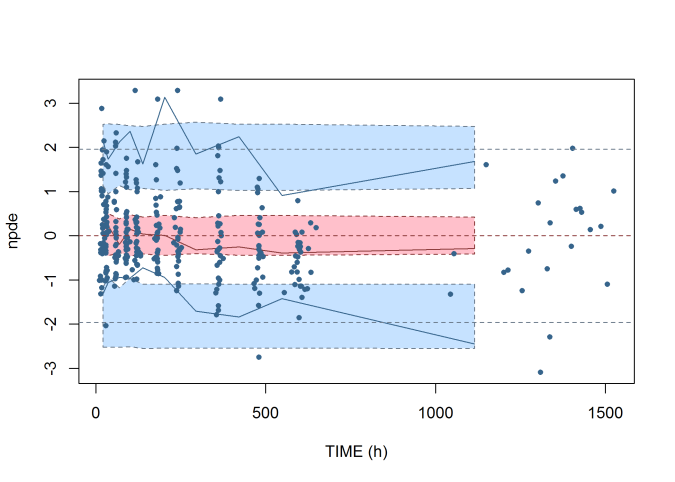

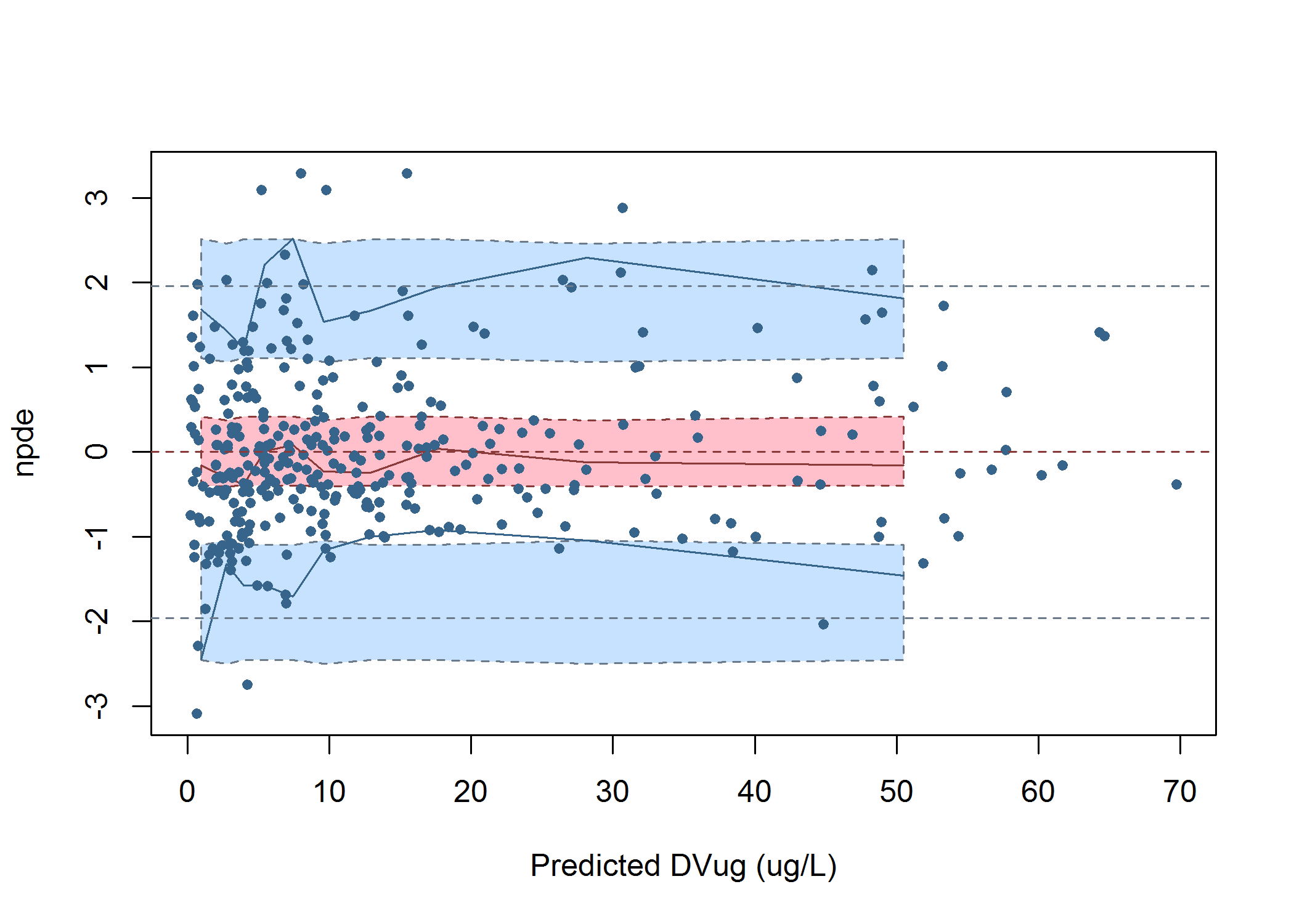
**

**Figure S3.** Visualization of the normalized prediction distribution error (NPDE) results for midazolam (E-G) showed that the mean NPDE of 0.016 was not significantly different from 0 (p>0.1, Wilcoxon signed rank test), and the variance for midazolam of 0.92 was not significantly different from 1 (p>0.1, Fisher variance test). A) Histogram of NPDEs, with the observed frequency of sample quantiles of the NPDEs (white bars), overlaid with the density of the standard normal distribution (blue bars). B) NPDE versus time, with the NPDE for each observation (dots), and the lines indicate the mean (red) and the 5^th^ and 95^th^ percentiles (blue) of the NPDEs, and the shaded areas are the simulated 95% confidence intervals of the NPDE median (red) and 5^th^ and 95^th^ percentiles (blue). C) NPDE versus predicted concentration, with dots and lines as described for panel B.

**Reference model Extrapolation model**

A E


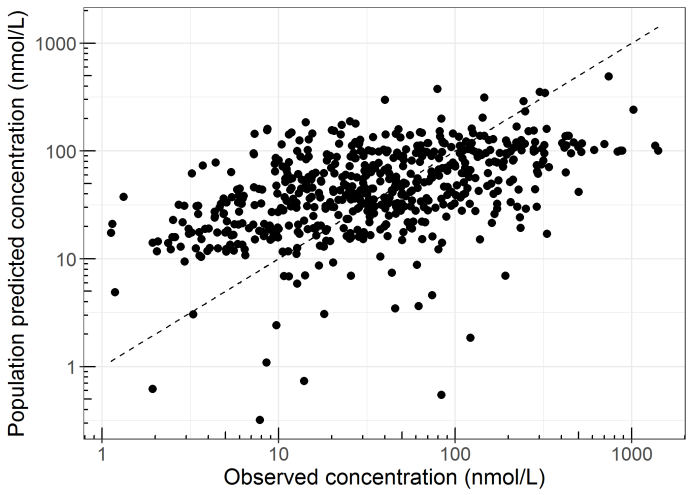

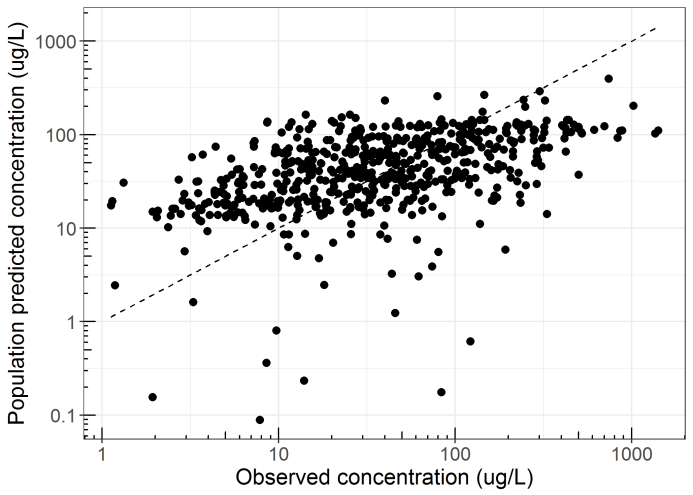


B F


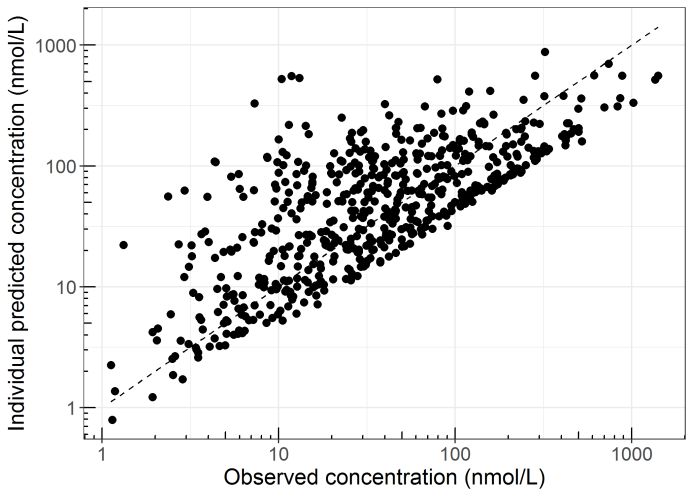

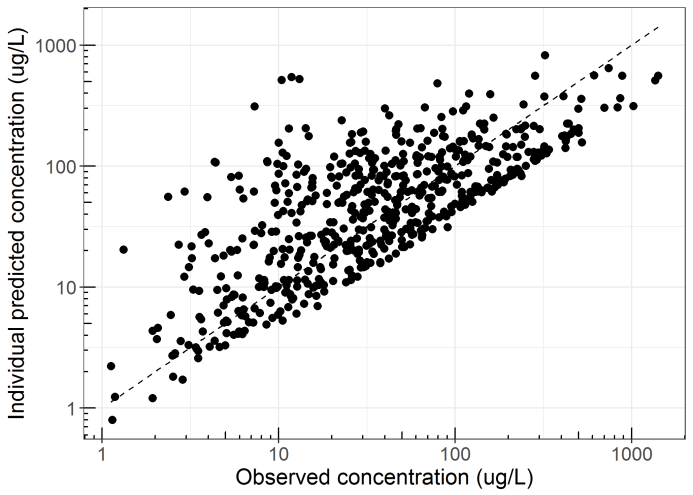


C G


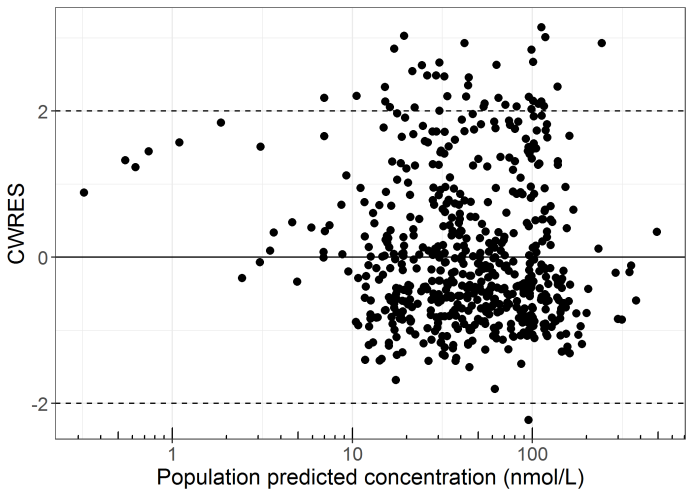

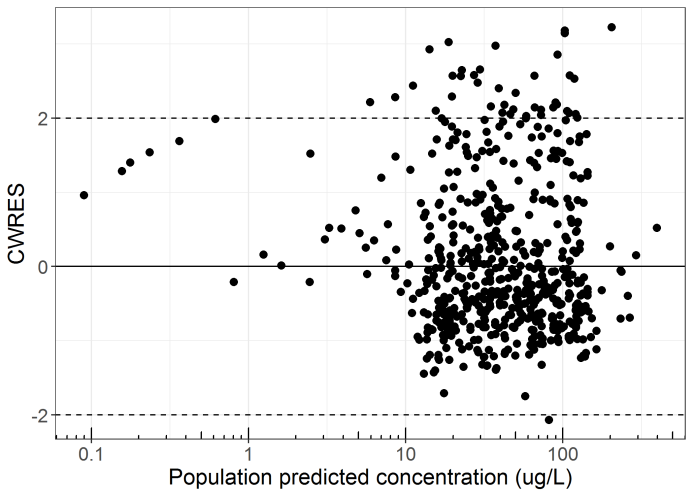


D H


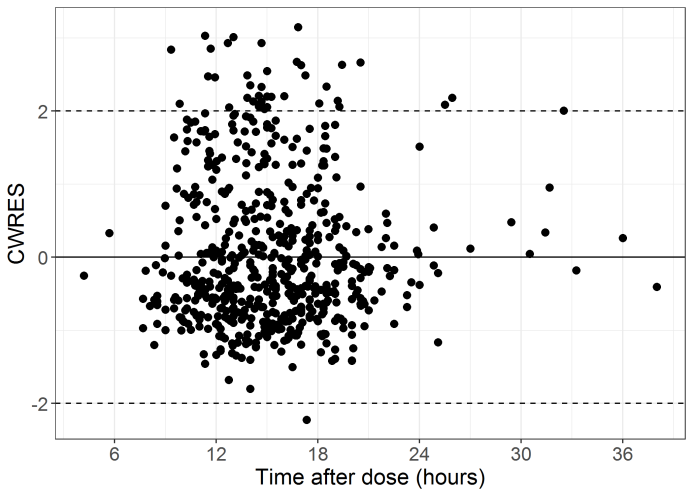

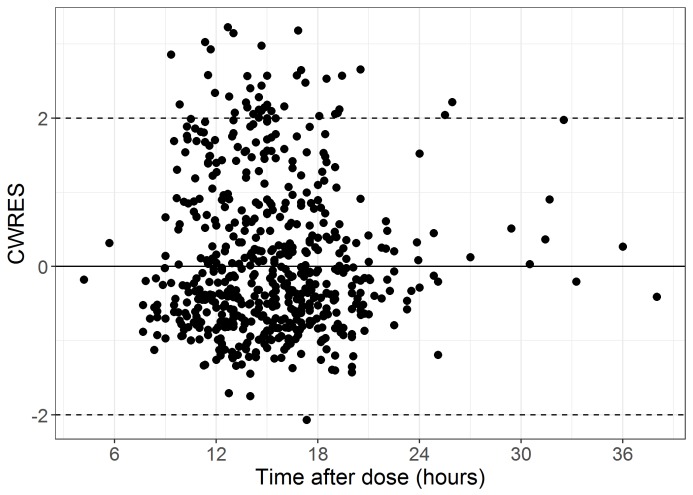


**Figure S4.** Goodness-of-fit plots for the two pediatric population PK models for the test drug (sildenafil). In the reference model clearance was estimated from the data (A-D) and in the extrapolation model the covariate model obtained with midazolam was used to describe the change in clearance in this population (E-H).

H

G

F

**Reference model Extrapolation model**

A D


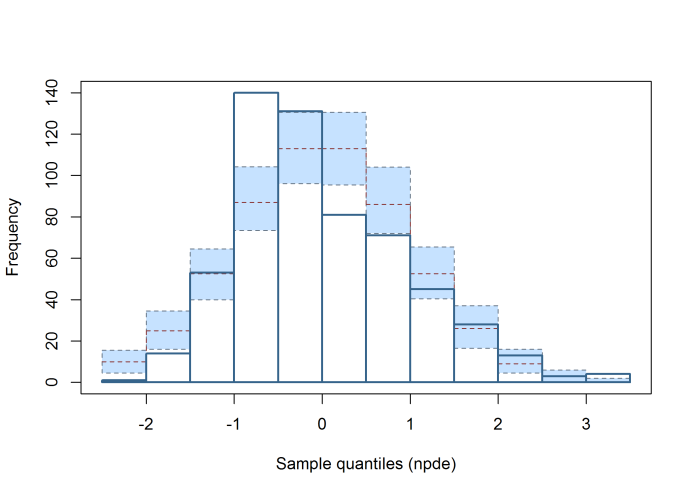

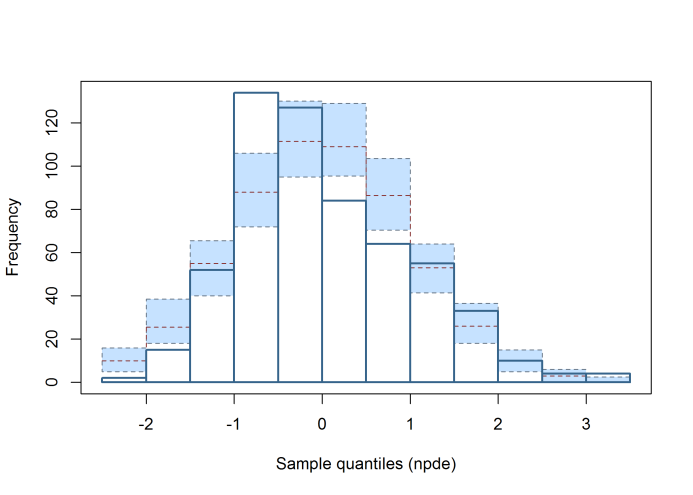


B E


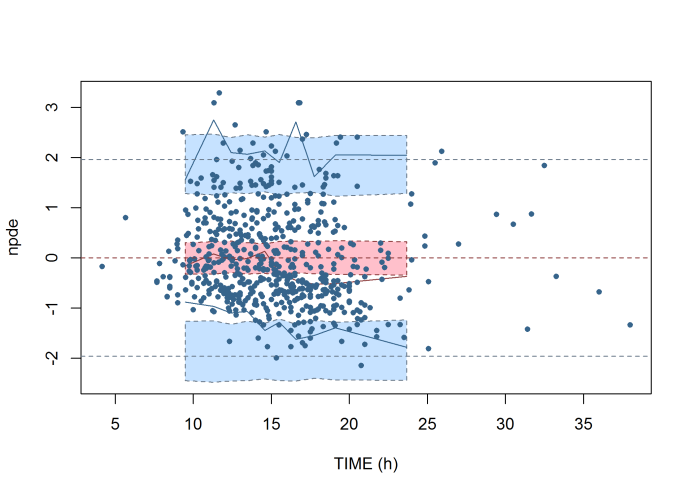

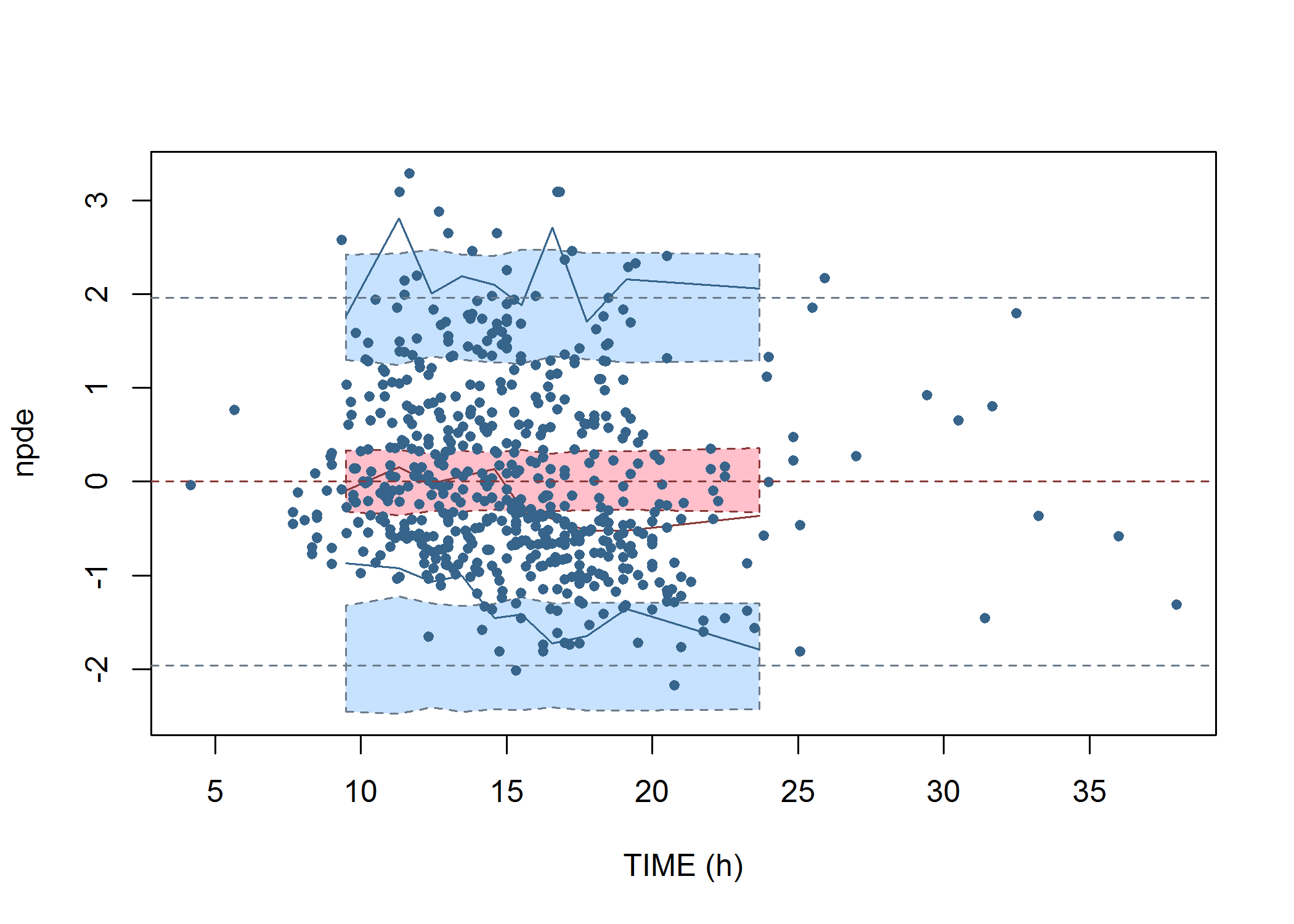


C F


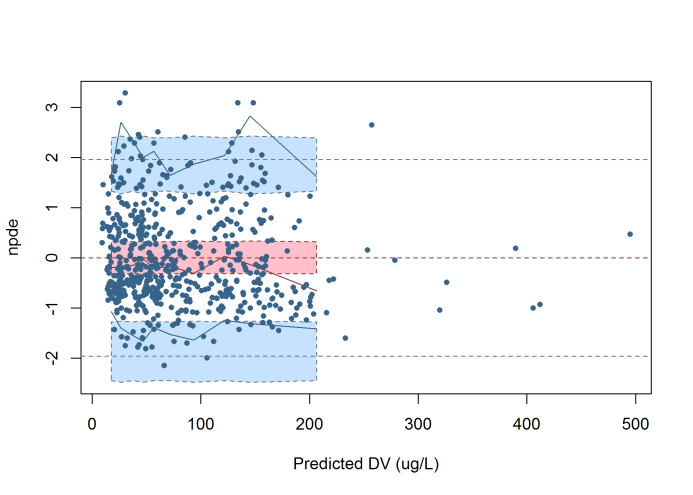

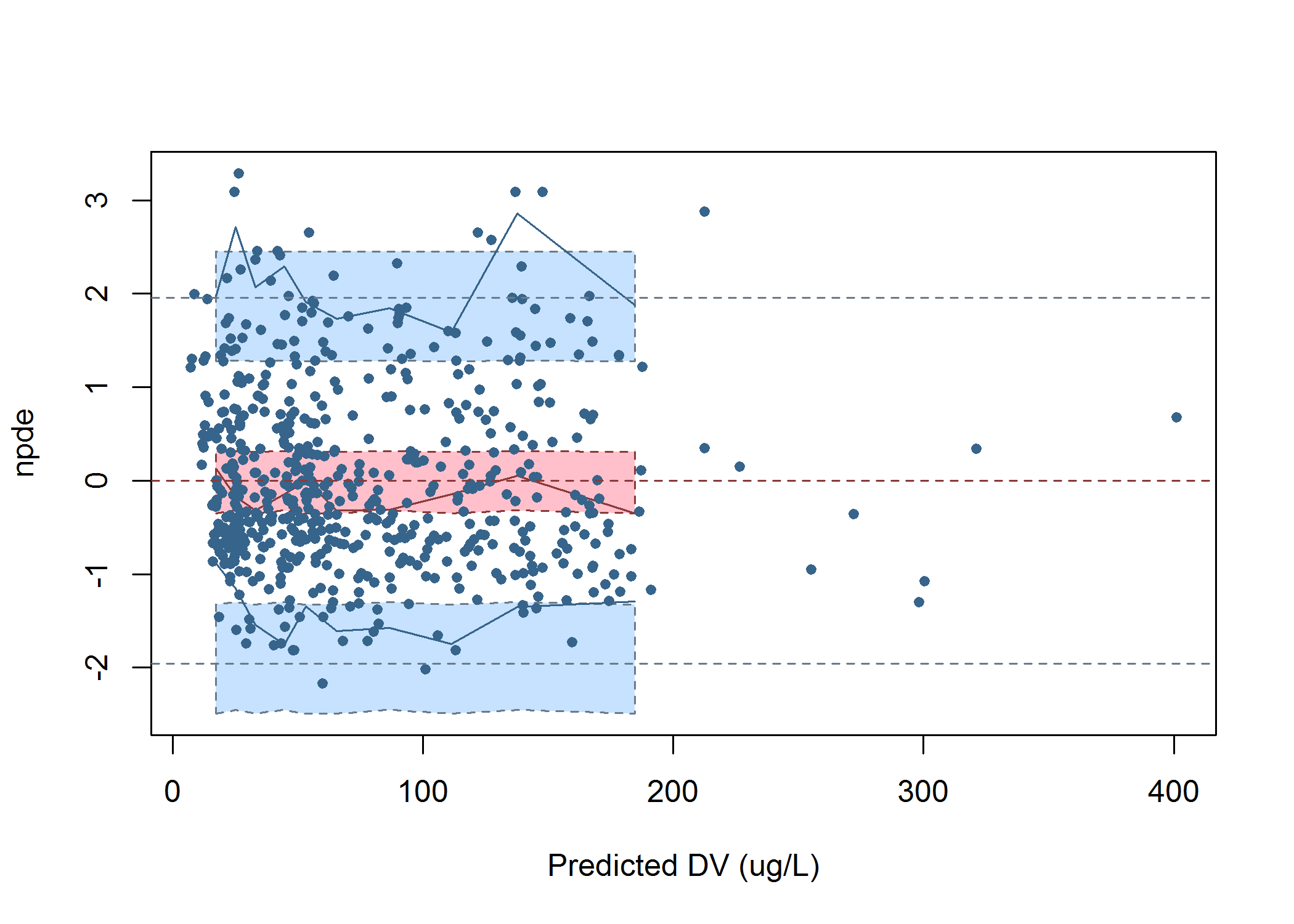


**Figure S5.** Visualization of the normalized prediction distribution error (NPDE) results for the sildenafil reference model (A-C) and the model based on between-drug extrapolation (D-F). The mean NPDE for the reference and extrapolated model were with 0.004 and 0.031, respectively, not significantly different from 0 (p>0.1, Wilcoxon signed rank test), and the variances were with 0.92 and 0.97, respectively, not significantly different from 1 (p>0.1, Fisher variance test). First row: Histogram of NPDEs, with the observed frequency of sample quantiles of the NPDEs (white bars), overlaid with the density of the standard normal distribution (blue bars). Second and third row: NPDE versus time and predicted concentration respectively, with the NPDE for each observation (dots), and the lines indicate the mean (red) and the 5^th^ and 95^th^ percentiles (blue) of the NPDEs, and the shaded areas are the simulated 95% confidence intervals of the NPDE median (red) and 5^th^ and 95^th^ percentiles (blue).

**References Supplementary Material**

33. Frechen S, Junge L, Saari TI, Suleiman AA, Rokitta D, Neuvonen PJ, et al. A semiphysiological population pharmacokinetic model for dynamic inhibition of liver and gut wall cytochrome P450 3A by voriconazole. Clin Pharmacokinet. 2013;52(9):763-81.

34. Lown KS, Thummel KE, Benedict PE, Shen DD, Turgeon DK, Berent S, et al. The erythromycin breath test predicts the clearance of midazolam. Clin Pharmacol Ther. 1995;57(1):16-24.

35. DrugBank Canada. Available from <https://www.drugbank.ca>. Accessed July 17th 2017.

36. Lennernas H. Clinical pharmacokinetics of atorvastatin. Clin Pharmacokinet. 2003;42(13):1141-60.

37. CVZ. College voor zorgverzekeringen Nederland; Farmacotherapeutisch Kompas (Dutch Pharmacotherapeutic Compass) 2013 [updated April 1, 2013. Available from: <http://www.fk.cvz.nl/>]. Accessed December 2017.

38. Rampe D, Roy ML, Dennis A, Brown AM. A mechanism for the proarrhythmic effects of cisapride (Propulsid): high affinity blockade of the human cardiac potassium channel HERG. FEBS Lett. 1997;417(1):28-32.

39. Heykants J, Hendriks R, Meuldermans W, Michiels M, Scheygrond H, Reyntjens H. On the pharmacokinetics of domperidone in animals and man. IV. The pharmacokinetics of intravenous domperidone and its bioavailability in man following intramuscular, oral and rectal administration. Eur J Drug Metab Pharmacokinet. 1981;6(1):61-70.

40. Simcyp (R) Simulator version 15.1. Certara, Sheffield, United Kingdom. Available from: https://www.certara.com/software/physiologically-based-pharmacokinetic-modeling-and-simulation/simcyp-simulator/. Accessed December 2017.

41. Bressler R, Bahl JJ. Principles of drug therapy for the elderly patient. Mayo Clin Proc. 2003;78(12):1564-77.

42. Zhao P, Zhang L, Grillo JA, Liu Q, Bullock JM, Moon YJ, et al. Applications of physiologically based pharmacokinetic (PBPK) modeling and simulation during regulatory review. Clin Pharmacol Ther. 2011;89(2):259-67.

43. Mehrotra N, Gupta M, Kovar A, Meibohm B. The role of pharmacokinetics and pharmacodynamics in phosphodiesterase-5 inhibitor therapy. Int J Impot Res. 2007;19(3):253-64.

44. Gruer PJ, Vega JM, Mercuri MF, Dobrinska MR, Tobert JA. Concomitant use of cytochrome P450 3A4 inhibitors and simvastatin. Am J Cardiol. 1999;84(7):811-5.

45. Benet LZ, Cummins CL, Wu CY. Unmasking the dynamic interplay between efflux transporters and metabolic enzymes. Int J Pharm. 2004;277(1-2):3-9.

46. Osmulski PA, Gaczynska M. Rapamycin allosterically inhibits the proteasome. Mol Pharmacol. 2013;84(1):104-13.

47. Doroshyenko O, Fuhr U. Clinical pharmacokinetics and pharmacodynamics of solifenacin. Clin Pharmacokinet. 2009;48(5):281-302.

48. Raucoules-Aime M, Kaidomar M, Levron JC, Le Moing JP, Goubaux B, Gugenheim J, et al. Hepatic disposition of alfentanil and sufentanil in patients undergoing orthotopic liver transplantation. Anesth Analg. 1997;84(5):1019-24.

49. Saari TI, Ihmsen H, Mell J, Frohlich K, Fechner J, Schuttler J, et al. Influence of intensive care treatment on the protein binding of sufentanil and hydromorphone during pain therapy in postoperative cardiac surgery patients. Br J Anaesth. 2014;113(4):677-87.

50. Tuteja S, Alloway RR, Johnson JA, Gaber AO. The effect of gut metabolism on tacrolimus bioavailability in renal transplant recipients. Transplantation. 2001;71(9):1303-7.

51. Piekoszewski W, Jusko WJ. Plasma protein binding of tacrolimus in humans. J Pharm Sci. 1993;82(3):340-1.

52. Matsushima H, Kamimura H, Soeishi Y, Watanabe T, Higuchi S, Tsunoo M. Pharmacokinetics and plasma protein binding of tamsulosin hydrochloride in rats, dogs, and humans. Drug Metab Dispos. 1998;26(3):240-5.

53. Dennison JB, Mohutsky MA, Barbuch RJ, Wrighton SA, Hall SD. Apparent high CYP3A5 expression is required for significant metabolism of vincristine by human cryopreserved hepatocytes. J Pharmacol Exp Ther. 2008;327(1):248-57.

54. Chan JD. Pharmacokinetic drug interactions of vinca alkaloids: summary of case reports. Pharmacotherapy. 1998;18(6):1304-7.

55. Knebel W, Gastonguay MR, Malhotra B, El-Tahtawy A, Jen F, Gandelman K. Population pharmacokinetics of atorvastatin and its active metabolites in children and adolescents with heterozygous familial hypercholesterolemia: selective use of informative prior distributions from adults. J Clin Pharmacol. 2013;53(5):505-16.

56. Dansirikul C, Morris RG, Tett SE, Duffull SB. A Bayesian approach for population pharmacokinetic modelling of sirolimus. Br J Clin Pharmacol. 2006;62(4):420-34.

57. Scholz J, Steinfath M, Schulz M. Clinical pharmacokinetics of alfentanil, fentanyl and sufentanil. An update. Clin Pharmacokinet. 1996;31(4):275-92.

58. Saeves I, Line PD, Bremer S, Vethe NT, Tveit RG, Meltevik TJ, et al. Tacrolimus exposure and mycophenolate pharmacokinetics and pharmacodynamics early after liver transplantation. Ther Drug Monit. 2014;36(1):46-53.

59. Tsuda Y, Tatami S, Yamamura N, Tadayasu Y, Sarashina A, Liesenfeld KH, et al. Population pharmacokinetics of tamsulosin hydrochloride in paediatric patients with neuropathic and non-neuropathic bladder. Br J Clin Pharmacol. 2010;70(1):88-101.

60. de Graaf SS, Bloemhof H, Vendrig DE, Uges DR. Vincristine disposition in children with acute lymphoblastic leukemia. Med Pediatr Oncol. 1995;24(4):235-40.

61. Hill KD, Sampson MR, Li JS, Tunks RD, Schulman SR, Cohen-Wolkowiez M. Pharmacokinetics of intravenous sildenafil in children with palliated single ventricle heart defects: effect of elevated hepatic pressures. Cardiol Young. 2016;26(2):354-62.

62. Szefler SJ, Pieroni DR, Gingell RL, Shen DD. Rapid elimination of quinidine in pediatric patients. Pediatrics. 1982;70(3):370-5.

63. Scott JR, Courter JD, Saldana SN, Widemann BC, Fisher M, Weiss B, et al. Population pharmacokinetics of sirolimus in pediatric patients with neurofibromatosis type 1. Ther Drug Monit. 2013;35(3):332-7.

64. Greeley WJ, de Bruijn NP, Davis DP. Sufentanil pharmacokinetics in pediatric cardiovascular patients. Anesth Analg. 1987;66(11):1067-72.

65. Jalil MH, Hawwa AF, McKiernan PJ, Shields MD, McElnay JC. Population pharmacokinetic and pharmacogenetic analysis of tacrolimus in paediatric liver transplant patients. Br J Clin Pharmacol. 2014;77(1):130-40.

66. Brocks DR, Mehvar R. Stereoselectivity in the pharmacodynamics and pharmacokinetics of the chiral antimalarial drugs. Clin Pharmacokinet. 2003;42(15):1359-82.
